# The effects of low-intensity blood flow restricted exercise compared with conventional resistance training on the clinical outcomes of active UK military personnel following a 3-week in-patient rehabilitation programme: protocol for a randomized controlled feasibility study

**DOI:** 10.1186/s40814-017-0216-x

**Published:** 2017-12-08

**Authors:** Peter Ladlow, Russell J. Coppack, Shreshth Dharm-Datta, Dean Conway, Edward Sellon, Stephen D. Patterson, Alexander N. Bennett

**Affiliations:** 1 Academic Department of Military Rehabilitation, Defence Medical Rehabilitation Centre (DMRC), Headley Court, Epsom, Surrey UK; 2 Department for Health, University of Bath, Bath, UK; 3 Imaging Department, Oxford University Hospitals, Oxford, UK; 4 School of Sport, Health and Applied Science, St Mary’s University, London, UK; 5 National Heart and Lung Institute, Faculty of Medicine, Imperial College London, London, UK

**Keywords:** Blood flow restriction, Musculoskeletal rehabilitation, Lower-limb, Muscle, Strength, Hypertrophy, Pain

## Abstract

**Background:**

A challenge for rehabilitation practitioners lies in designing optimal exercise programmes that facilitate musculoskeletal (MSK) adaptations whilst simultaneously accommodating biological healing and the safe loading of an injured limb. A growing body of evidence supports the use of resistance training at a reduced load in combination with blood flow restriction (BFR) to enhance hypertrophic and strength responses in skeletal muscle. In-patient rehabilitation has a long tradition in the UK Military, however, the efficacy of low intensity (LI) BFR training has not been tested in this rehabilitation setting. The aims of this study are to determine (1) the feasibility of a randomised controlled trial (RCT) investigating LI-BFR training in a residential, multidisciplinary treatment programme and (2) provide preliminary data describing the within and between-group treatment effects of a LI-BFR intervention and a conventional resistance training group in military personnel.

**Methods:**

This is a single-blind randomised controlled feasibility study. A minimum of 28 lower-limb injured UK military personnel, aged 18 to 50 years, attending rehabilitation at the UK Defence Medical Rehabilitation Centre (DMRC) will be recruited into the study. After completion of baseline measurements, participants will be randomised in a 1:1 ratio to receive 3 weeks (15 days) of intensive multidisciplinary team (MDT) in-patient rehabilitation. Group 1 will receive conventional resistance training 3 days per week. Group 2 will perform twice daily LI-BFR training. Both groups will also undertake the same common elements of the existing MDT programme. Repeat follow-up assessments will be undertaken upon completion of treatment. Group 2 participants will be asked to rate their pain response to LI-BFR training every five sessions.

**Discussion:**

The results will provide information on the feasibility of a full-scale RCT. Recommendations for an adequately powered study to determine the efficacy of LI-BFR training during in-patient rehabilitation can then be made. The study may also provide insights into the potential effectiveness of LI-BFR training as a novel exercise modality to induce muscle adaptations in the absence of high mechanical loading of the lower-limb.

**Trial registration:**

ISRCTN Reference: ISRCTN 63585315 dated 25 April 2017.

## Background

The maintenance of adequate skeletal muscle is crucial for maintaining the ability to undertake activities of daily living, ambulation, falls avoidance and general health [1]. Disuse of skeletal muscle, often associated with musculoskeletal (MSK) injury, can lead to relatively rapid and progressive atrophy; shortening of muscle fibres, decreased oxidative capacity, and reduced muscle compliance [2]. It is widely acknowledged that muscle atrophy can prolong the duration of MSK rehabilitation, increase the cost to health care providers and prevent optimal recovery [3]. Thus, strategies to increase or maintain muscle tissue across the lifespan are crucial for overall health and quality of life.

The goal of the surgical and rehabilitative team focuses on the safe return of a patient to their previous level of function. MSK rehabilitation can be considered in terms of the appropriate integration and progression of the following broad exercise components: endurance, flexibility, proprioception, balance, joint and soft tissue mobility, speed and power [4]. Strength training is most closely associated with improvements in functional ability during rehabilitation [5]. Therefore, maximising the potential for adaptations in muscle strength is a crucial factor in the progression of any MSK exercise rehabilitation programme. A significant challenge lies in designing optimal rehabilitation programs that facilitate both neurological and muscular adaptations whilst accommodating biological healing and patient safety [4]. Historically, it has been widely accepted that to elicit significant gains in muscle hypertrophy and strength requires loads equivalent to at least 70% of an individual’s 1 repetition maximum (1RM) for a given movement [6, 7]. For people undergoing musculoskeletal injury rehabilitation, heavy-load resistance training can be contraindicated [2] or they are limited by their symptomatic impairment, including pain and immobility, to attain the recommended heavier-loads [8]. Therefore, patients with MSK injuries are often advised to reduce their training load, potentially limiting the desired muscular response to treatment.

In recent years, research demonstrates that the use of blood flow restriction (BFR) combined with low-load resistance exercise (20–40% 1RM) can enhance the morphology and strength response in human muscle tissue [9]. BFR is typically achieved via a pressurised cuff [10], tourniquet [11] or elastic banding [12]. The external pressure applied to the proximal portion of the upper or lower extremities should be low enough to maintain partial arterial inflow into the muscle, but high enough to occlude venous return from the muscle [13]. During periods of immobilisation, the application of BFR alone has been shown to reduce muscular atrophy [14]. However, to optimise muscular development, BFR must be combined with an exercise stimulus (aerobic conditioning or resistance training), with the greatest muscle strength and morphological responses achieved when BFR is combined with resistance training [2].

When supervised by experienced practitioners, low-intensity BFR (LI-BFR) has been shown to be a safe and effective tool to improve strength and function in athletes [15], the elderly [16, 17] healthy adults [18] and during MSK rehabilitation [19–24]. Additional benefits reported with LI-BFR training, is the potential for increases in muscle hypertrophy and strength in muscles located proximal to the applied pressure (i.e. muscles not direct under BFR) as a result of pre-fatigue of the muscles below the cuff [25]. It is possible that this additional muscle stimulus proximal to the cuff (e.g. in the Gluteus Maximus muscle during a squat or leg press) may further enhance physical function and accelerate progression during MSK rehabilitation. Adverse events to acute sessions of LI-BFR have been reported and primarily include delayed onset of muscle soreness, numbness, fainting/dizziness and bruising [26]. There have also been case study reports of rhabdomyolysis [27, 28] and retinal occlusion [29] in the literature. However, when appropriately supervised, LI-BFR is widely acknowledged as a safe mode of exercise in healthy adults [30].

It is proposed that the metabolic stress associated with BFR and the mechanical tension of the load lifted act synergistically to mediate numerous secondary mechanisms, all of which stimulate autocrine and/or paracrine actions to facilitate muscle growth [31]. These proposed mechanisms include muscle cell swelling [32], elevated systemic hormone production [33, 34], intramuscular anabolic/anti-catabolic signalling [35–37], increased fast twitch fibre recruitment [38] and the production of reactive oxygen species (ROS) [39, 40] and its variants, including i) nitric oxide for its influence on vascular responses [41, 42] and ii) some heat shock proteins [36, 39]. However, in the absence of research demonstrating a causal link, any suggested associations between BFR training and subsequent muscle growth are purely speculative.

**Fig. 1:**
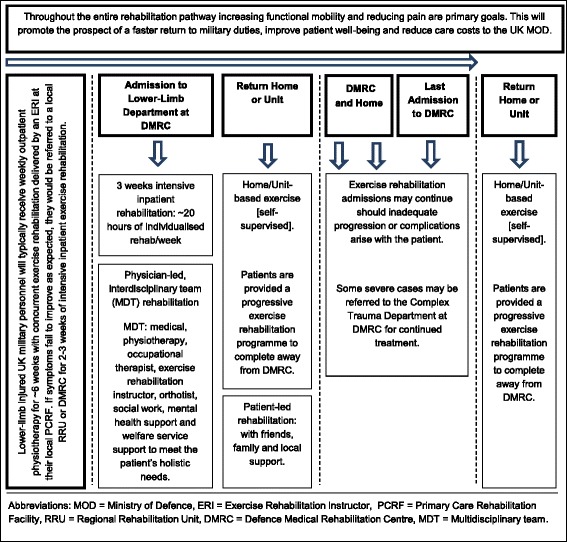
Lower limbs rehabilitation pathway at DMRC, Headley Court

There is increasing evidence for the practical and beneficial use of BFR training as a clinical MSK rehabilitation tool [43]. Any intervention that speeds the progression of MSK rehabilitation, whilst exercising at lower relative training loads, is of interest not only to the rehabilitation and sports medicine communities, but the wider community health services. In military populations, the majority of injuries occur in the lower limb [44]. In a cohort of 6608 British Army recruits, during a 26-week period of initial military training, the overall incidence of musculoskeletal injuries was 48.6% [45]. There is a large economic and operational cost associated with lower-limb MSK injury. Soldiers injured during basic training, field exercise, sport etc. may be unable to deploy on operations, whilst soldiers injured during deployment may not be fit to return to active duty [44]. The Centre for Lower-Limb Rehabilitation at the UK Defence Medical Rehabilitation Centre (DMRC), Headley Court routinely treats and manages a large variety of lower-limb musculoskeletal disorders (See Fig. 1 for a diagrammatic model of the rehabilitation pathway). These typically include, but are not limited to, overuse injuries (e.g. patellofemoral pain, tendinopathy, early osteoarthritis, and exertional lower-limb pain), post-surgical injuries (e.g. soft-tissue and ligamentous reconstruction), bone fractures, and hip and groin pain.

Of particular interest, in relation to the UK military model of exercise rehabilitation (Fig. 1), is the evidence of muscular hypertrophy and strength demonstrated with high frequency (twice a day) BFR training in as little as 6 days [46] and 12 days [47] of training. The effect of this novel training method is yet to be explored across the UK Defence Medical Services (DMS). Therefore, the purpose of this study is to assess the feasibility of LI-BFR training in a heterogeneous group of lower-limb injured military personnel, under the conditions provided during a 3-week intensive residential rehabilitation centre. This includes measuring the hypertrophic and strength response but also reporting any potential adverse events, monitoring compliance and the pain response over time and whether frequent daily use of this clinical tool is feasible in a busy MDT clinical rehabilitation setting where other potentially conflicting clinical priorities exist. This protocol describes the design and analysis plan for a randomised controlled feasibility study.

## Methods/design

The primary aim of this study is:

1. To assess the acceptability, feasibility and adverse events associated with implementing a high-frequency LI-BFR intervention in a 3-week intensive residential MDT rehabilitation setting.
2. To assess the feasibility of a future definitive RCT by assessing participant eligibility, monitoring recruitment and retention rates, group allocation acceptance and adherence to the intervention.

The secondary aim is to compare the effects of LI-BFR training against conventional resistance training on cross-sectional area (CSA) and volume of the quadriceps and hamstring muscle groups and muscle strength in UK military personnel undergoing lower-limb injury rehabilitation. Changes in relevant musculoskeletal variables of treatment routinely measured as part of the standard UK military rehabilitation care pathway will also be assessed. This includes walk/run assessment, balance, pain perceptions and compliance to the exercise rehabilitation programme.

### Study design

**Fig. 2:**
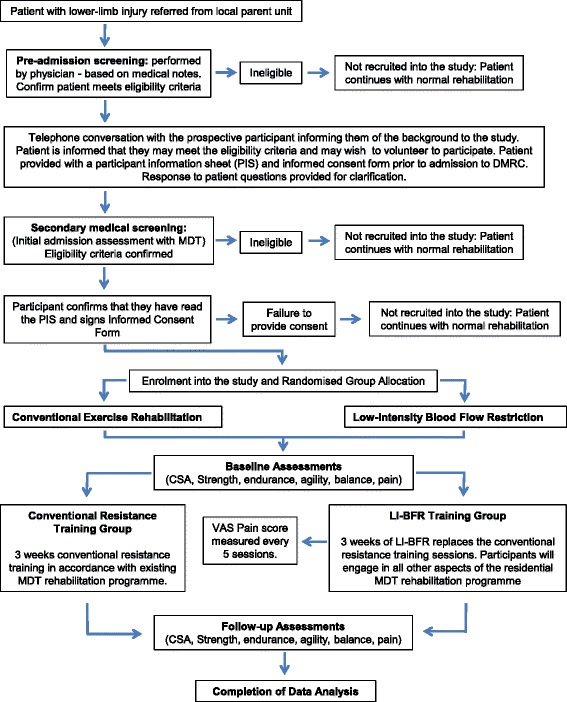
Study design

This is a parallel group, two-arm, assessor-blinded randomised controlled feasibility study. It is a two (group) by two (time) repeated measures design. Outcome measurements will be assessed at baseline and 3-weeks. The study protocol has been developed in accordance with the Standard Protocol Items: Recommendations for Interventional Trials (SPIRIT) guidelines [48]. The overall study design is illustrated in Fig. 2.

### Setting

The study will be conducted at a specialist UK military rehabilitation centre.

### Ethics

The study was reviewed and approved by the UK Ministry of Defence (MOD) research ethics committee (study reference protocol number: 442/MODREC/13). Any requirement for protocol modifications will be submitted for authorisation to the MOD research ethics committee.

### Study participants

We will recruit a minimum of 28 participants aged 18-50 years admitted for treatment to the Centre for Lower-Limb Rehabilitation (Defence Medical Rehabilitation Centre (DMRC), Headley Court, UK) with a MSK injury of the lower limb. Table 1 describes the inclusion and exclusion criteria. These criteria are designed to recruit a heterogeneous group of lower-limb injured patients who are able to engage in load bearing conventional resistance training, but do not have a functional status allowing a return to full operational military duties. A significant majority of patients admitted to DMRC for rehabilitation are male. In a ‘time limited’ period allocated for data collection, we could not predict the number of females available for recruitment into the study. Therefore, because the primary purpose of this study is to assess the feasibility and not the effectiveness of the LI-BFR intervention, for ease of administration and logistics, we chose to recruit males only. If feasible, any future full-scale RCT will recruit both male and female participants.

**Table 1:** Study inclusion and exclusion criteria

Inclusion criteria
1. Male
2. 18 to 50 years of age
3. Serving regular UK Armed Forces personnel
4. Lower limb injury (e.g. patellofemoral pain, ACL reconstruction, ankle injury, projectile/blast related injury)
5. Referred to Defence Medical Rehabilitation Centre (DMRC), Headley Court for treatment.
6. Present with a level of function enabling engagement in conventional load bearing exercise rehabilitation confirmed by clinical assessment findings and recent training history.
7. Unable to return to active duty due to physical impairment and occupational limitations (e.g. medical downgrading).
Exclusion Criteria
1. Female
2. History of cardiovascular disease (hypertension, peripheral vascular disease, thrombosis/embolism, ischaemic heart disease, myocardial infarction)
3. History of the following musculoskeletal disorders: rheumatoid arthritis, avascular necrosis or osteonecrosis, severe osteoarthritis
4. History of the following neurological disorders: Peripheral neuropathy, Alzheimer’s disease, amyotrophic lateral sclerosis, multiple sclerosis, Parkinson’s disease, stroke, mild or severe traumatic brain injury
5. Chronic or relapsing/remitting gastrointestinal disorders such as inflammatory bowel diseases, irritable bowel syndrome or gastrointestinal infections within 28 days of screening.
6. Acute viral or bacterial upper or lower respiratory infection at screening
7. Moderate or severe chronic obstructive pulmonary disease (COPD)
8. Amputation to the lower or upper extremity
9. Known or suspected lower limb chronic exertional compartment syndrome (CECS) (tourniquet raises intra-compartmental muscle pressure)
10. Achilles or patella tendinopathy (slow heavy resistance or eccentric exercise programme prescribed as evidence-based for confirmed tendinopathy diagnosis)
11. ACL surgery within the last 4 weeks
12. Surgical insertion of metal components in lower limbs (may affect MRI results)
13. History of any of the following conditions or disorders not previously listed: diabetes, fibromyalgia, active cancer, severe obesity (i.e., body mass index greater than 35 kg/m^2^), diagnosed mental illness (e.g. PTSD, depression, anxiety)
14. Current or previous use of any drugs known to influence muscle mass or performance within previous 6 months
15. Elevated risk of unexplained fainting or dizzy spells during physical activity/exercise that causes loss of balance

### Randomisation and blinding

Potential participants will be referred from their parent military unit by a physiotherapist or medical officer. Prior to admission to DMRC, the case records of individual patients scheduled for a lower-limb rehabilitation course will be reviewed by a specialist rehabilitation consultant (SDD). Those who meet the preliminary inclusion criteria will be contacted via telephone by a member of the research team to discuss their possible inclusion in the study. Potential participants will be sent an information pack consisting of the patient information sheet (PIS) and an accompanying consent form. Upon admission to DMRC Headley Court, potential participants will undertake a comprehensive musculoskeletal examination by a specialist consultant and experienced musculoskeletal physiotherapist where a secondary screening will confirm the patient’s eligibility to enter the study. Participants meeting the eligibility criteria, who have read and understood the PIS and volunteer to participate will return a signed informed consent form, before being randomly assigned to one of the two study groups. A block randomisation method will be used to randomise participants into groups that result in equal sample sizes. Our decision to employ a simple form of block randomisation is because (a) we want to ensure an equal number of participants are assigned to each group during a finite, time-limited period for data collection, (b) we already have a homogenous participant group with standardised prognostic factors, and (c) we will not be undertaking formal statistical testing. A plain language statement will inform participants that they have an equal chance of receiving the LI-BFR or conventional resistance training intervention. A sealed envelope will be opened to reveal group allocation by an independent administrator not involved in the recruitment, treatment or assessment of study outcomes. Group allocation will be documented and communicated to the supervising therapists by the independent administrator. Prior to the study, all treating staff will have received a briefing on the randomisation process and specific intervention for each treatment group, in line with the study protocol. It is not possible to blind participants to treatment allocation in this study. The clinical staff supervising both groups will be, by necessity, un-blinded. We will use trained blinded outcome assessors to measure and record the outcome scores in this study. A diagrammatic description of the study design can be found in Fig. 2.

### Combined LI-BFR and resistance training protocol—current guidelines

Scott et al. [9] has provided evidence-based guidelines on optimal muscle hypertrophy and strength responses to LI-BFR training. This includes using cuff widths (~6 to 13.5 cm) for the legs and resistance training at 50 to 80% of occlusion pressure taken at rest; a detailed description of the technique used to establish occlusion pressure is provided in a later section (LI-BFR Group). BFR can be used with low-intensity exercise (~20–40% of 1 RM), utilising 50 to 80 repetitions per exercise, with occlusion maintained during inter-repetition rest periods of 30 to 45 s. Both single and multi-joint exercises can provide benefit and clinical populations are advised to complete two to three training sessions per week; however, training twice each day with BFR is possible. These guidelines will form the foundations of our LI-BFR training protocol.

### Generic lower-limb rehabilitation intervention

The total duration of treatment is 3 weeks, utilising 15-days of specific MDT exercise rehabilitation (Monday to Friday). There is no follow-up period, as the aim is to assess the feasibility and effects of the intervention(s) during the period of in-patient rehabilitation. A summary of the DMRC lower-limb MDT rehabilitation programme and treatment components is provided at Fig. 1 and Table 2, respectively. This generic treatment approach has been described elsewhere by Coppack et al. [49]. All participants will receive individualised programmes focussing on improving range of motion, balance, aerobic conditioning, manual therapy and education sessions. The generic MDT programme will be common to both groups with only the resistance training or LI-BFR intervention individualised to each participant dependent on group allocation. Additional information regarding each of these components is provided below.

**Table 2:** Components of generic in-patient rehabilitation programme

Treatment modality	Treatment content	Treatment goals	Typical number of sessions per week
Individualised Exercise: led by ERI (45–60 min)	Strengthening exercises, active range of motion exercises, functional balance drills, gait drills, progressive coordination drills, non-weight-bearing aerobic/endurance exercise	Restore strength of major muscle groups of the lower-limb, improve core strength, increase joint range of motion, improve balance and neuro-muscular control, and improve muscle endurance.	3–4
Individualised physiotherapy (30–60 min)	Manual therapy techniques, muscle activation and timing patterns, active and passive range of motion exercises, advice on home exercise, gait re-education training	Improve quality and timing of movement, improve muscle strength, reduce pain, increase joint range of motion, induce relaxation, promote normal walking gait.	1–3
Group Exercise: led by ERI (45–60 min)	Group based circuit training that primarily involves high repetition muscular strengthening exercises targeting the whole body. May also include minor team games, recreational therapy, foam rolling, stretching, motor control, running re-education, cv	The same as the Individual exercise sessions, but also the promotion of group cohesion and social support	12
Hydrotherapy/swimming (30/45 min)	Non-weight-bearing aerobic exercise, strengthening exercises, active range of motion exercises, self-paced recreational swimming, progressive/assisted weight-bearing exercise and activity	Improve muscle strength, improve aerobic capacity, increase joint range of motion, improve confidence in weight bearing, induce relaxation, and promote enjoyment and fun.	1 hydro 3 swim
Individualised occupational therapy session (30–60 min)	Relaxation techniques, postural re-education, cognitive behavioural therapy techniques, self-help coping strategies, pain management.	Induce relaxation, promote behavioural change, control pain, correct/improve poor posture	0–3
Patient education (60 min)	Coping with pain, benefits of exercise, joint protection, anatomy and pathology of their lower-limb injury, nutrition.	Goal setting, activity modification, reduction of pain, promote behavioural change, weight management, improve knowledge of treatment options, improve ability to relax, improve knowledge of self-help techniques	4

### Stretching and range of motion exercise

Static and active stretching and foam-roller techniques will be employed to maintain the range of motion (ROM) required for optimal function. This forms part of routine clinical practice within lower-limb rehabilitation in the UK military.

### Neuromuscular control and functional balance exercise

Balance and proprioceptive exercises will be included to restore deficits and re-establish neuro-motor control. Progression will be applied by increasing the complexity and difficulty of the exercise, by reducing the base of support, adding dynamic movements on unstable surfaces and increasing the range through which the movement is performed. Support for neuromuscular training in lower-limb rehabilitation has been reported in the literature [50].

### Aerobic exercise

Participants will undertake light to moderate aerobic conditioning over the intervention period. In addition to the general health benefits conferred by aerobic exercise, moderate joint loading has been shown to be beneficial for joint health because of mechanosensitive chondroprotective pathways [51]. No study has described the optimal dose of aerobic exercise for patients undergoing lower-limb rehabilitation in terms of intensity, volume and duration. In this study, the supervising ERI will determine the nature of aerobic exercise (walking, cycling, swimming, cross-trainer) and progression in intensity based on individual examination finings and patient response to exercise.

### Manual therapy

Manual therapy techniques will be used to modify the quality and range of motion of soft tissue structures, and assist with pain relief. The manual therapy intervention will be prescribed individually for each participant on the basis of the physical examination findings, from a list of techniques including, trigger point massage, passive joint mobilisation, distraction and sustained stretches [49]. These techniques are commonly used in the management of injured military personnel at DMRC and delivered by their respective MSK physiotherapist.

### Education

Educating the patient on factors surrounding their treatment and the importance of regular exercise is a key component of the rehabilitation process at DMRC to optimise patient adherence to home or work-based exercise programmes. Education and advice will be a focus of the intervention and will include information on diagnosis and aetiology of their injury, rationale for treatment, the benefits of exercise, joint protection and activity modification strategies, pain management, coping with acts of daily living (sitting, driving, sleeping, work) and the importance of increasing physical activity levels in everyday life [49]. Unsupervised home-based prescription of BFR has not been investigated and therefore not recommended in the UK Defence best practice guidelines. Instead, the LIBFR group will be prescribed a conventional resistance training programme to perform upon discharge from the rehabilitation centre.

### Intervention group 1—LIBFR group

#### Determining limb occlusion pressure

The participant’s limb occlusion pressure will be determined during a one-off procedure prior to commencing the LI-BFR training programme. The participant lies in a semi-recumbent supine position on a treatment couch and a contoured 66 × 10 cm or 90 × 10 cm width blood pressure cuff (Schuco TourniCuff, Schuco International, Watford, UK) is placed around the most proximal part of each thigh. The length of the cuff (60 or 90 cm) will be selected based on the participant’s thigh girth, thereby ensuring sufficient overlap of the inflatable regions of the cuff to provide occlusion to the lower-limb. The cuff size will be recorded and then used for the entirety of that participant’s LI-BFR training programme. The posterior tibial or dorsalis pedis pulse is found with a MD2 vascular Doppler probe (Huntleigh Healthcare Ltd., Cardiff, UK). The wide contoured tourniquet will rapidly be inflated using a PTSii portable tourniquet system (Delfi Medical Innovations, Vancouver, Canada) to a pressure of 250 mmHg [52] so that the audible pulse is lost. If the pulse is not lost at 250 mmHg, then the cuff will be inflated in increments of 10 mmHg until the pulse is abolished. The cuff will be deflated in increments of 10 mmHg until the pulse is found again. This provides an estimate to the nearest 10 mmHg. The cuff will then be fully deflated. After a 30 s rest, the cuff will be inflated to the estimate pressure + 10 mmHg. It will then be deflated more slowly in increments of 5 mmHg, so the occlusion pressure can be determined to the nearest ±5 mmHg. 60% of this limb occlusion pressure is calculated to be used as the tourniquet pressure during the LI-BFR intervention [9]. Subsequent limb blood occlusion pressure assessments will be performed on day 7 and the final training day to measure potential changes in occlusion pressure over time. Any measured changes in pressure on day 7 will not influence the cuff pressure used during the trial.

### LI-BFR exercise protocol

**Fig. 3:**
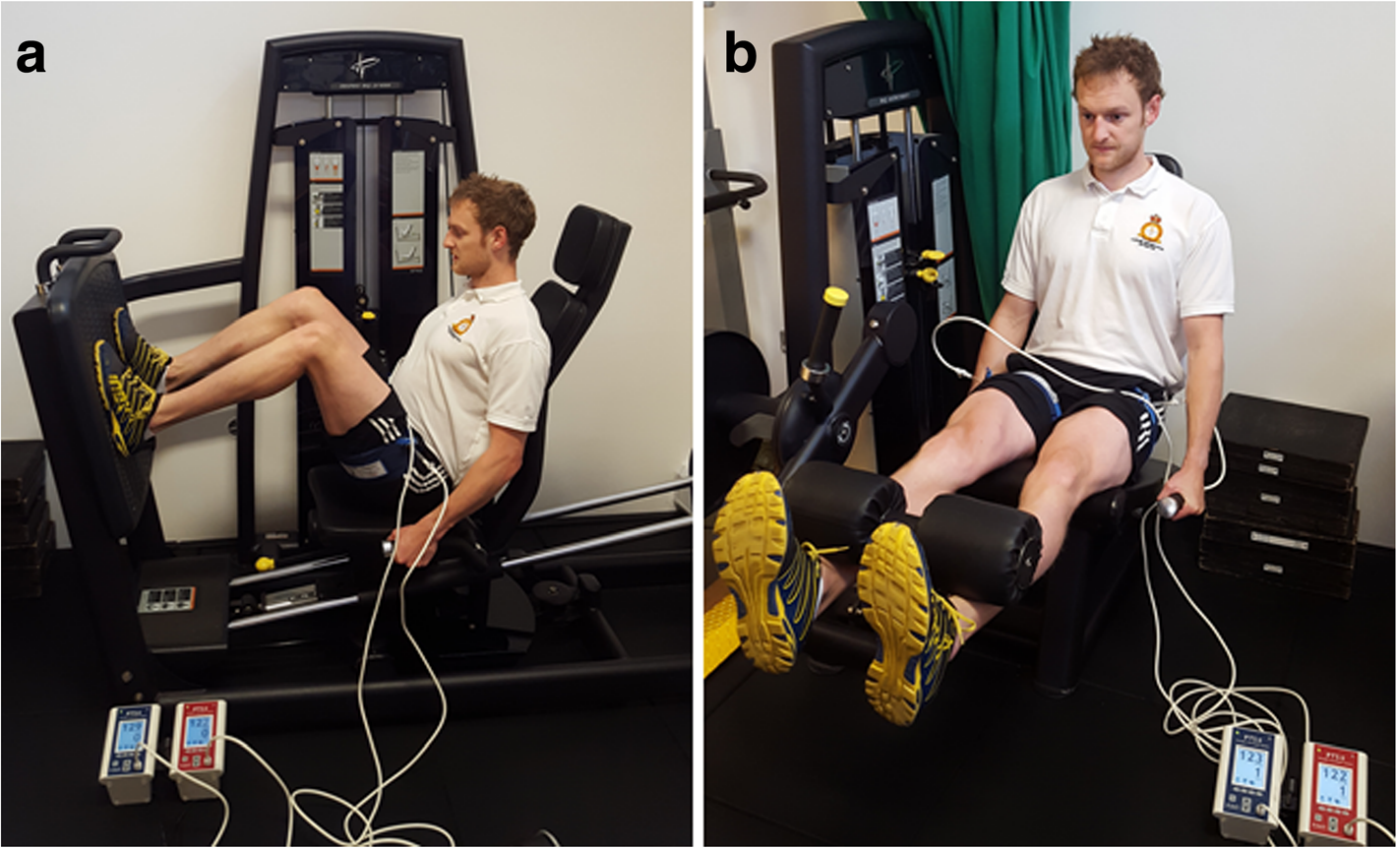
Low intensity blood flow restriction (LI-BFR) exercises: **a** leg press, **b** knee extension

Participants will perform low intensity resistance training combined with blood flow restriction using two exercises in sequence: (1) bilateral leg press using a Leg Press Machine (Pulse Fitness, Congleton, UK), and (2) bilateral knee extensions using a Leg Extension Machine (Pulse Fitness, Congleton, UK) (see Fig. 3).

Prior to exercise, each participant will undergo a standardised 5 min progressive warm-up on a stationary bike (Wattbike Ltd., Nottingham, UK). Wide contoured blood pressure cuffs will then be placed around the most proximal part of each thigh and inflated using a PTSii portable tourniquet system to 60% occlusion pressure. Participants will then perform 4 sets of 30, 15, 15 and 15 repetitions (75 repetitions in total) at 30% of their predicted 1RM, assessed during their 5RM muscle strength assessments with an inter-set interval of 30 s (see secondary outcome measures below for a detailed description of the 5RM muscle strength assessment protocol). To ensure consistency of lifting between patients, a metronome is set at 60 bpm, with 1 s for the completion of the concentric phase, no pause followed by a 1 s eccentric phase of the lift (1:0:1 tempo).

The inflation pressure will be maintained for the duration of the exercise and then deflated for 3 min to allow the participant a small recovery period and to move to the next exercise/equipment station. It will then be re-inflated to the target 60% occlusion pressure and the second exercise station (leg extension) will commence. Therefore, the length of time induced to restricted blood flow will be 4 min per exercise and 8 min per training session. Training will be performed twice daily, in the morning (between 08:00 and 09:30) and afternoon (between 14:00 and 15:30) from Monday to Thursday and once on Friday morning. Over the 15 days of rehabilitation, MDT clinical assessments will be carried out on the first and last day of an admission. This allows a maximum of 23 LI-BFR training sessions over a total period of 13 treatment days. Daily LI-BFR sessions will always be separated by interludes of at least 5 h. Assuming patients adapt over the 3 week residential programme, the 1RM is expected to increase and therefore even when exercising at 30% 1RM, we would expect to increase the weight lifted by small increments (e.g. 2.5 kg increase per week). Any increase in weight lifted will be at the discretion of the exercise rehabilitation instructor (ERI) and participant, and training load (the number of repetitions and load lifted) for each session will be monitored and recorded accordingly.

### Intervention 2 conventional resistance training

Participants will engage in conventional load bearing resistance training, typically consisting of four sets of three exercises (deadlift, back squat and lunges) performed three times per week. A gradual and timely exercise progression is determined by the ERI based upon participant feedback, re-assessment and individual response to training. It should be noted that despite an abundance of information on the implementation of strength and conditioning principles with healthy participants, investigation regarding the application of these principles in rehabilitation programmes is lacking [53]. Therefore, a relatively conservative initial dosage is chosen that should allow a short period of adaptation whilst controlling for pain, thereby promoting exercise adherence. Patients will be educated on correct movement patterns before loading to volitional fatigue. Repetitions per set are typically six to eight and tailored to the individual needs of the patient with rest intervals between each set approximately 3 min. The dosage for strengthening exercises in this protocol aims to meet the ongoing challenge of designing treatment programmes that facilitate neurological and muscular adaptations whilst concurrently accommodating biological healing, recovery, and the safety of the patient. The justification for the initial dosage of four sets of six to eight repetitions takes account of the evidence suggesting pain provoked by exercise has been shown to reduce adherence to exercise in rehabilitation programmes [54]. The load lifted is a reflection of their best effort taking into account each individual’s respective limitations due to injury.

### Outcome measures

All outcome measures are to be assessed at baseline and upon completion of 3 weeks in-patient rehabilitation. Pain response and training load will be recorded over several time points during the BFR intervention (Fig. 2). The authors acknowledge that due to the likely interference effect of a pain response, performing a ‘maximum effort’ physical task in a lower-limb injured cohort is unlikely to yield a true measure of MSK performance. It is more accurate to describe outcome scores as providing an ‘indication’ of participant performance and progression. We will highlight this as a potential weakness in our study but feel this is a challenge in the measurement of muscle force/strength in all MSK injury research. All outcomes measured will be implemented and recorded based upon a best effort at the time of assessment.

### Descriptive data

Personal and demographic characteristics including age, stature, body mass, body mass index (BMI), gender, duration of symptoms, previous injuries, previous treatments, medication use, military occupation, duration of military service, smoking and drinking habits will be obtained during the initial participant assessment or obtained via electronic medical notes using the Defence Medical Information Compatibility Program (DMICP).

### Primary outcome measure: feasibility and acceptability of LI-BFR intervention

The main focus of this study is feasibility and acceptability for recruitment, retention and measurement of the LI-BFR intervention. Recruitment rates will be measured as the rate of eligible participants invited and consenting into the study. Acceptability of allocation/randomisation procedures will be assessed by examining the reasons for drop-out in any discontinuing participants and by comparing attrition rates between groups. Session adherence rates will be recorded to provide a measure of compliance with the intervention. Strengths, weaknesses and safety of LI-BFR intervention will be assessed by qualitative interviews with the project supervisor, lead exercise rehabilitation instructor, participant feedback and examination of any adverse event reports. We will also aim to provide an estimate of the recommended sample-size for a fully-powered future RCT [55].

### Secondary outcome measure: muscle cross sectional area (CSA) and volume

Thigh muscle cross sectional area (CSA) (cm^2^) and volume (cm^3^) will be assessed prior to and 1 day following the subject’s rehabilitation training programme, using magnetic resonance imaging (MRI) with a GE Sigma scanner 1.5 T (General Electric, Wisconsin, USA), in accordance with the method previously described by Abe et al. [56]. A T1-weighted, spin-echo, axial plane sequence will be obtained with continuous transverse images from the greater trochanter to the lateral condyle of the femur with a 1.0 cm slice thickness and no inter-slice gap. If this distance exceeds 50 cm, two separate sequence acquisitions will be required with a triglyceride skin marker used for sequence co-registration. The MRI data will be transferred onto a study laptop computer for analysis by a UK Defence Consultant Radiologist, using specially designed image analysis software (TomoVision Inc., Montreal, Canada) [57]. For each slice on the injured limb, quadriceps and hamstring muscle compartment CSA (cm^2^) will be measured and muscle compartment volumes calculated (cm^3^). In participants with bilateral pathology, one leg will be randomly selected for muscle analysis. The coefficient of variation (CV) for this measurement technique has been demonstrated to be less than 1% [56]. Repeat MRI assessments will be performed 24 h after the participant’s final LI-BFR training session.

### Secondary outcome measure: muscle strength

Unilateral muscle strength will be assessed using a dynamic 5 RM test performed on the knee extension and leg press machines (Pulse Fitness, Congleton, UK). Following a general warm-up on a stationary bike for 5 min, subjects will perform a 10-repetition warm-up on the knee extension and leg press respectively. An initial resistance is set by the supervising ERI based upon the result of a clinical assessment, pain intensity and participant feedback. The resistance is then adjusted and test repeated until the participant is unable to complete five repetitions. The baseline 5RM is then recorded by the supervising ERI. Subjects will have 3 min rest between each attempt. This procedure follows established and widely used guidelines [58].

### Secondary outcome measure: functional performance assessment

Functional ability will be assessed using the following standardised outcome measures used in the current best-practice care pathway at DMRC. These tests will be conducted and recorded by an experienced supervising therapist.

### Isometric muscle strength proximal to the cuff

**Fig. 4:**
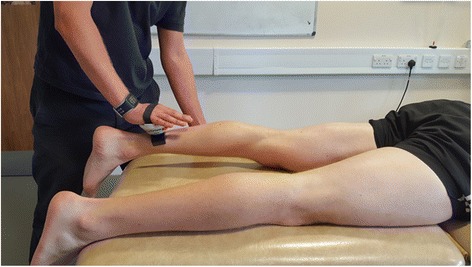
Hip extension strength measure using microFET-2 hand-held dynamometer (HHD)

Unilateral isometric muscle strength will be measured at the start and end of the 3 weeks. Measurements will be taken using a wireless digital microFET2 hand-held dynamometer (Hoggan Scientific LLC, Drapper, UT, USA) for hip extension only (see Fig. 4). Isometric hip extension strength will measure muscular adaptations proximal to the cuff. Participants will be tested on a clinical examination couch using procedures often applied in the clinical setting [59]. This test was chosen as isometric loading induces less stress on the musculoskeletal system than eccentric loading (‘break-test’), which is a key consideration when testing individuals with a physical injury [60]. A long lever arm will be utilised during the test to ensure the tester’s strength exceeds the isometric force applied by the participant. The examiner will apply resistance in a fixed position whilst the participant exerts a 5 s isometric maximal voluntary contraction (MVC) against the dynamometer and the examiner. Participants will perform four consecutive attempts with a 30 s recovery between attempts. Strength measures will be reported as Newtons (N). The highest value will be used for analysis purposes. Good interrater reliability (ICC 0.76–0.79) and low test-retest variation (< 10%) has been demonstrated for the HHD measurement technique in measuring isometric muscle strength [59].

### Multi-stage locomotion test (MLFT)

The objective of this test is to assess the participant’s maximal walk/run distance [61, 62]. The test requires the participant to walk/run on a 20 m track at gradually increasing speeds until they are unable to continue due to an increase in symptoms. Speed is controlled by paced-auditory cues accompanied by recorded verbal instructions. The test will be terminated once a patient fails three consecutive attempts at reaching the designated marker at the sound of the audible cue. Total distance covered in metres will be recorded.

### Figure of 8 test

This test measures the participant’s agility and acceleration/deceleration ability on a flat surface. Within the limitations of their injury, each participant will be required to complete 3 laps of a figure of 8 walk/run at maximal speed, in accordance with established guidelines [63].

### Y-balance test

**Fig. 5:**
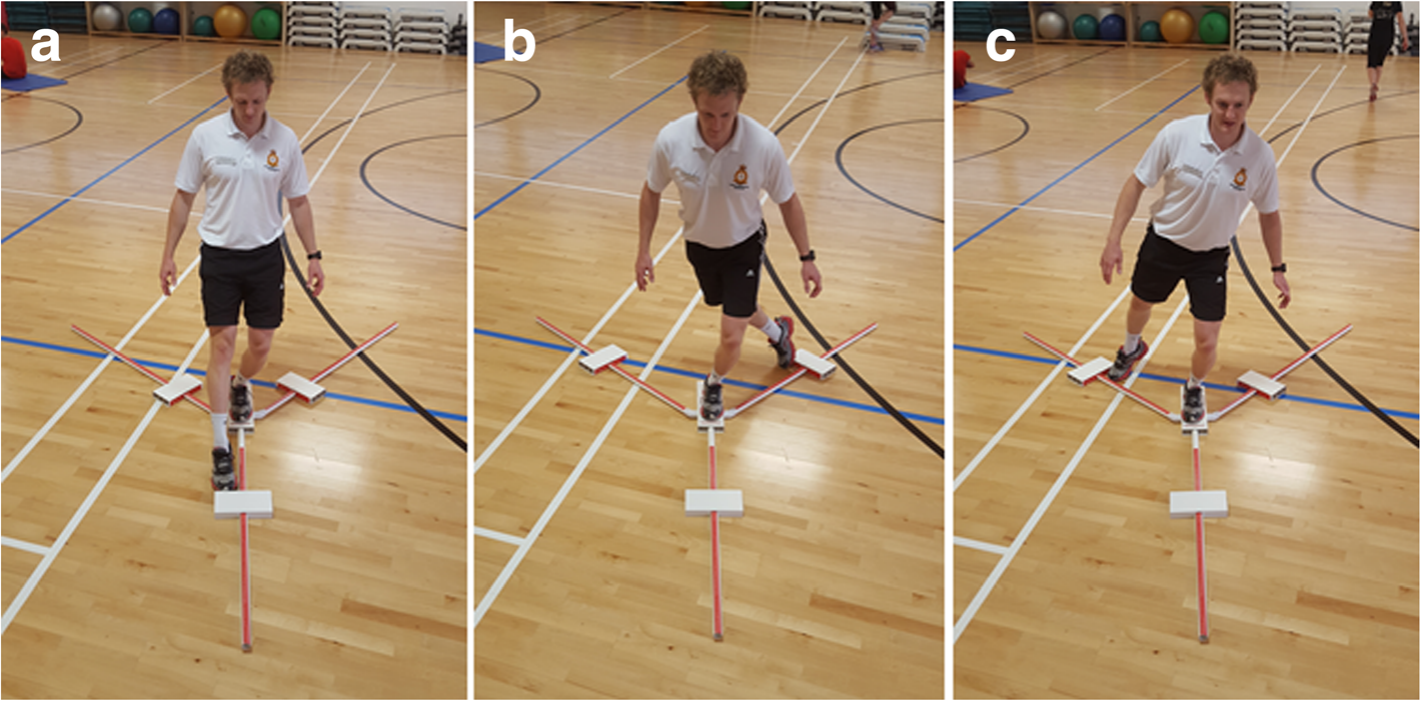
Y Balance test: **a** anterior, **b** posteriomedial, **c** posteriolateral reach direction

This test developed by Plisky et al., [64] assesses lower-body balance and flexibility using the Y-Balance test kit®. Standing through a single supporting limb on the test kit, the participant will reach with the free limb as far as possible along three lines positioned in anterior, posteromedial and posterolateral directions on each leg (see Fig. 5a–c). The test is currently used as a measure of postural control in patients undergoing UK military lower-limb rehabilitation.

### Pain perception

It is recognised that BFR training can cause mild muscle discomfort. A visual analogue scale (VAS) will be used to measure pain intensity. The VAS uses a 100 mm horizontal line anchored by the terms ‘no pain’ (0) and ‘worst possible pain’ (100). The VAS response format has shown good internal consistency, is easy to understand, is in wide clinical use, and has been sufficiently evaluated in clinical trials [65]. It is useful in this prospective study of twice daily LI-BFR training sessions to record pain scores in the injured limb. This will be recorded immediately prior to starting the exercise, during the exercise and then 5 min post-exercise. This will be repeated every five BFR training sessions to monitor how pain response changes over time to the BFR intervention.

### Sample size

As this is a pilot-feasibility study we will not perform a formal sample size calculation determined by statistical assumptions and tests. Sample size recommendations for pilot randomised controlled trials will be followed [66] and will aim for a minimum of 12 participants in each study arm providing full data. Given the time constraints for data collection, we intend to recruit 14 participants into each treatment group (i.e. total sample size of 28). We consider this will provide sufficient data to adequately address the aims of this feasibility study and provide useful information on key issues such as recruitment, retention and acceptability of the LI-BFR intervention. This pragmatic preliminary sample will inform a power analysis for a full-scale RCT.

### Statistical analyses

Data generated from this pilot study will help inform a future fully-powered RCT by testing the study procedures. We will not use feasibility trial data to formally test for between-group differences, and the analysis will be of a descriptive nature. Therefore, no statistical analyses will be performed for the pilot data. Descriptive statistics (mean, standard deviation (SD), counts (percentage)) will be used to summarise eligibility, consent, randomisation, adverse events, retention, completion and intervention adherence rates. Description of participant demographic and baseline characteristics will be compared and simple tabulation of this pilot data will be presented. The 95% confidence intervals will be calculated to inform a sample size for a future definitive RCT. All participants will be included in the analysis. This statistical analysis of the pilot data will be exploratory only as our sample size will not allow for a definitive analysis. We will recommend progression to a full study application if minimum criteria are reached in key feasibility aims and objectives. These criteria will include a minimum 80% of target participant recruitment over a 6-month period, and a minimum 80% completion of the LI-BFR intervention and outcome measurement.

### Adverse events

All clinical and research staff will receive a brief detailing the procedures for identifying and reporting safety issues including the use of project adverse events forms. Information on any unexpected adverse events deemed to be related to study participation will be collected and reported to the chief investigator within 24 h of its occurrence. Reporting of safety incidents will be duplicated using existing DMRC clinical health and safety reporting procedures and in accordance with the principles of good clinical practice (GCP). It is not anticipated that there will be any risk to study participants.

## Discussion

Optimising the recovery of UK Military personnel suffering MSK injury is of critical importance and has been highlighted as a priority for research by the UK Defence Medical Services. The premise that the use of BFR combined with low-load resistance exercise can enhance the strength response in human muscle tissue may have implications for the rate of recovery in patients undergoing injury rehabilitation. We describe the rationale and design of a feasibility study for the introduction of an LI-BFR intervention into a residential, MDT rehabilitation programme for military patients suffering a variety of lower-limb MSK injuries. To date, this is the first study to establish the preliminary effects of LI-BFR on the muscle volume, strength measures and functional capacity of military personnel undergoing intensive, in-patient rehabilitation. Furthermore, the recruitment methods, safety, intervention adherence and acceptability of a twice daily LI-BFR intervention are essential feasibility components that need to be understood prior to embarking on a fully powered randomised controlled trial. Therefore, the findings from this feasibility study will inform a full-scale trial to determine the effectiveness of LI-BFR during in-patient, MDT rehabilitation. If feasible, military and civilian health care providers could consider LI-BFR as a cost-effective, practical rehabilitation modality to induce muscle adaptation in the absence of high mechanical loading of the lower-limb. The results of the trial will be published when they are available.

## Abbreviations

BFR: Blood flow restriction
CECS: Chronic exertional compartment syndrome
CSA: Cross-sectional area
DMRC: Defence Medical Rehabilitation Centre
DMS: Defence Medical Services
ERI: Exercise Rehabilitation Instructor
GCP: Good clinical practice
LI-BFR: Low intensity blood flow restriction
MDT: Multidisciplinary team
MRI: Magnetic resonance imaging
MSK: Musculoskeletal
MSLT: Multi-stage locomotion test
MVC: Maximal voluntary contraction
PIS: Participant information sheet
RM: Repetition maximum
ROM: Range of motion
ROS: Reactive oxygen species
VAS: Visual analogue scale
